# Supplementary material for: Characterization of Alternative Splicing Events in Porcine Skeletal Muscles with Different Intramuscular Fat Contents
Source: Biomolecules. 2022 Jan 18;12(2):154. doi: 10.3390/biom12020154 (PMC8961525; doi:10.3390/biom12020154)
Supplement: Supplementary file 1 [file biomolecules-12-00154-s001.zip › Table S1 Primers used in this study.pdf]

Table Primers used in the study

Gene Name/ID	Sequence (5'-3')
Novel isoform validation	
PB. 4.1712.1	F: GCCCTGTTGGTTGTGAGATG
	R: CTGAGGACGTTGGCTTTGTG
PB. AEMK02000400.1.1.1	F: CATTGCCATGCTATCACCG
	R: CACCTCTTTCGCCATCCAC
PB. 13.2154.1	F: TGGGTCATAGGGCATGTTTA
	R: GCAGGCTGGTATTGTTTGG
PB. 12.56.2	F: GCTGCCGGTCTTGCATCTTCA
	R: AGCCTGCGATACATGACCCCA
PB. 6.2001.1	F: GTATGTGGAAATGTATTTGACGA
	R: GAGTGCATGTGGGTAGGTG
PB. 10.295.4	F: CGTCCACCACCTGGCTCAC
	R: CAACCACAAGCACAGAAGTAGG
PB. 5.869.1	F: GTGTCTCAGGTGCTGCAATCA
	R: CTTCCCAGCCCTGTGTCCA
PB. 4.1070.1	F: CCTACTACTTCCCTTCTTCACCGA
	R: CTGTTGACCTTTCACCCTCCTT
PB. 18.2.1	F: GCCAAGCTCAGATGGTGTTCA
	R: CATCTTCCCTCAGACATACTCC
PB. 14.55.1	F: GAACGGAGGGTTATGTGGTCTG
	R: GCACTGAATCTTGGGTGTTTGGT
PB. 16.236.1	F: CTGTAGCAAAGGGCAAGTGTCA
	R: CCTGTAGGACTTTGAGAAACGCA
PB. 5.899.1	F: TGAGTCTAAGTTTGCGGGAAT
	R: ACACTCTCCAAAATGTCTTACG
ENSSSCG00000005316 -	F: AGGATGCCCAGGAGAAACT
ENSSSCG00000029441	R: AGGCTGGTATTCTGGGTGTG
ENSSSCG00000029219 -	F: GGGGCTACAGCTTCACCA
ENSSSCG00000010190	R: GCCAGCACCTTCTTCTTCATC
ENSSSCG00000028355 -	F: CTGGGTCCATAGCGATTAG
ENSSSCG00000031903	R: CTGTTGGAATGGGGTTTAG
Alternative splicing validation	
ENSSSCG00000001988	F: CGTGCTCTGCGGAGTTATTGG
	R: TGACCTGGAAGAACTTGGCATC
ENSSSCG00000051428	F: GCGGCTCTGGAGAAGTGTAA
	R: GGAGGCAATTCAAAGTCAAA
ENSSSCG00000023272	F: AGTTCCCCGGAGCAGGCT
	R: GAGGTCGTTCTCCGTGTTGT
ENSSSCG00000006388	F: CAGAAGGATGACCCAAGACGG
	R: GAAGTAGGGCCAGGCTGAGCT
ENSSSCG00000024827	F: AGGGCAAGAGTGTTGTGGT

	R: TTCTCCTGGAAGTGGTCAT
ENSSSCG00000013282	F: CACGATCCCTCCTTGGCG
	R: ATGCCCGTGGCGATGAG
Differential alternative splicing validation	
ENSSSCG00000040419	F: GGTGGACGCCGTGTAATG
	R: GTGCGAGAGTGGGTATCAGC
ENSSSCG00000022112	F: AGTGAGAAGAGCAATTCCCTG
	R: GCCGTAGCTGTGGTTAAAAG
Real-time PCR validation	
ACSL1	F: GTAGTGAGCGATTGTTCAGCGTTTG
	R: GCGAGAGGCAAGAAAGAGATCAGAG
PDLIM7	F: CGGCAGTAATGGGAAGAC
	R: ATAGCGCACATCATAGCAG
ACADM	F: GATGAAGCTACCAAGTATGC
	R: CATAATAGGTATTTCGGCG
RCAN1	F: ATCTCACCTCCTGCCTCG
	R: CTTCATTCTTTCCATCTCGT
ZFAND6	F: TCCCAGAAGCTCAGTCAA
	R: GGCTGCTGTGCCGTATC
AMPD1	F: GCCTCGCCTGTCAATCT
	R: AATGTTCTCCAGCATCTTTC
SLC38A2	F: GCCTGTTCGAAGCTGTCTA
	R: TGTGCCGATGCCCAAAT
